# Seizure characteristics and neurological outcomes in pediatric anti-NMDAR encephalitis: a cohort study from Southern China

**DOI:** 10.1007/s10072-025-08660-3

**Published:** 2026-01-09

**Authors:** Haixia Zhu, Wenlin Wu, Chi Hou, Wenxiao Wu, Yanping Ran, Lianfeng Chen, Kelu Zheng, Yani Zhang, Yiru Zeng, Yang Tian, Yuanyuan Gao, Bingwei Peng, Xiuying Wang, Yinting Liao, Xiaojing Li, Wen-xiong Chen

**Affiliations:** 1 Department of Neurology, Guangzhou Women and Children’s Medical Center, Guangzhou Medical University, Guangzhou, 510623 China; 2 Department of Behavioral Development, Guangzhou Women and Children’s Medical Center, Guangzhou Medical University, Guangzhou, China

**Keywords:** anti-NMDAR encephalitis, Seizure, Status epilepticus, Outcome, Children

## Abstract

**Objectives:**

This study aimed to investigate seizure characteristics, treatment and outcome of pediatric anti-NMDAR encephalitis in southern China.

**Methods:**

Clinical data of pediatric patients diagnosed with anti-NMDAR encephalitis were were retrospectively analyzed. Neurological disability and seizure severity were assessed using the mRS score and NHS3 scale, respectively.

**Results:**

Among 119 children with anti-NMDAR encephalitis, 74.8% (89/119) presented with seizures, with SE in 19.3% (23/119). The relapse rate was significantly higher in patients with seizures during the acute stage (19.1%, 17/89) compared to those without seizures (3.3%, 1/30) (*P* = 0.041). The mean NHS3 score for seizures during the acute stage was 7.9 ± 0.9. Patients with > 3 seizure episodes had higher mRS pre-immunotherapy, required longer hospital stays and received more courses of intravenous methylprednisolone (IVMP) or intravenous immunoglobulin (IVIG) courses and were more likely to require Rituximab. EEG abnormalities were observed, including background slowing in all cases, epileptic discharges (37.0%), and extreme delta brush in 8.4%. 53.9% (48/89) of patients with seizure received anti-seizure medications (ASMs), 72.9% (35/48) of whom were treated with monotherapy. Seizure remission was achieved in 95.8% (46/48) within one year; 25.0% (12/48) discontinued ASMs after a median of 6.0 months (IQR 3.5, 15.0 months) without seizure recurrence, while 75.0% (36/48) continued ASMs at the last follow-up, with 5.6% (2/36) still experiencing seizures despite CSF anti-NMDAR antibodies turned negative. Patients with status epilepticus (SE) had longer diagnostic delays and higher pre-immunotherapy mRS scores, required more IVIG courses, but showed a similar prognosis compared with non-SE patients.

**Conclusion:**

Seizures are common in pediatric anti-NMDAR encephalitis; patients with more than three seizure episodes had higher mRS pre-immunotherapy and required more intensive treatment; Approximately half of the patients required ASMs, with 95.8% achieving remission within one year. The relapse rate was significantly higher in patients with seizures during the acute stage compared to those without seizures. Patients with SE had higher mRS pre-immunotherapy and required more IVIG courses, but their prognosis was similar to that of non-SE patients.

## Introduction

Anti-N-methyl-D-aspartate receptor (NMDAR) encephalitis is the most common type of autoimmune encephalitis with seizure and status epilepticus (SE) being frequent manifestations [1–4]. Although substantial literature exists on anti-NMDAR encephalitis [3, 5], there is a paucity of data specifically addressing seizure characteristics and long-term outcomes in pediatric patients. Additionally, no universal guidelines exist for the management of seizures in children with this condition. Although few large-scale clinical studies have focused on the characteristics and outcomes of seizure events in pediatric anti-NMDAR encephalitis, and the long-term management of seizures remains controversial. In light of this, we conducted an analysis of the clinical characteristics, electroencephalograph (EEG) findings, treatment, and prognosis of seizure and status epilepticus in children with anti-NMDAR encephalitis from one of the National Children’s Medical centers in southern China. This study aims to provide insights to improve the evaluation, treatment selection, and long-term prognosis for these patients.

## Methods

### Patients

Children diagnosed with anti-NMDAR encephalitis between October 2014 andOctober 2020 in the department of neurology of Guangzhou Women and Children’s Medical Center were included. Informed consents were obtained from all legal guardians of the children included in the study. This study was approved by the Ethics Committee of Guangzhou Women and Children Medical Center.

### Data collection

Clinical features included demographic data, clinical manifestations, seizure type, laboratory investigation, EEG, and brain magnetic resonance imaging (MRI). Neurological disability was assessed using a modified Rankin Scale (mRS) [6]. Seizure severity was evaluated according to the the Chalfont-National Hospital seizure severity scale (NHS3) [7]. The mRS and NHS3 were assessed before and after immunotherapy treatment and at the final follow-up. A good long-term prognosis was defined as mRS score ≤ 2, while a poor long-term prognosis was defined as mRS score > 2 [8]. Cerebrospinal fluid (CSF) pleocytosis was defined as white cell count>15/µl in our center. All patients underwent tumor screening including computed tomography, ultrasound and MRI.

### Inclusion criteria

Patients aged younger than 18-year-old and met the anti-NMDAR encephalitis diagnostic criteria proposed by Graus et al. [9] were involved.

### Exclusion criteria

Patients were excluded if they were primary schizophrenia and cognitive behavior abnormalities secondary to an intracranial infection, drugs, poisoning, brain trauma, genetic and metabolic diseases and psychological diseases, or developed anti-NMDAR encephalitis after herpes simplex encephalitis or other viral encephalitis or diagnosed with epilepsy prior to the onset of anti-NMDAR encephalitis.

### Definition

The acute stage of anti-NMDAR encephalitis was defined as the first 3 months after the onset of encephalitis [9, 10]. The seizure type classification was based on clinical symptoms assessed by neurologists and was according to the International League Against Epilepsy 2017 criteria [11]. Status epilepticus diagnosed by the 2015 International League Against Epilepsy SE diagnostic criteria [11–14]. Refractory SE is characterized by persistent seizures after adequate and standardized treatment with benzodiazepines and other anti-seizure medications (ASMs), requiring general anesthesia, with refractory and poor prognosis [15]. An episode of seizure was defined as a discrete clinical seizure event.

### Antibodies test

Anti-NMDAR IgG in serum and CSF were detected by fixed cell-based assay (EUROIMMUN, Lübeck, Germany). These antibodies test was performed by an independent medical agency during acute attacks or follow-up visits. These methods had been reported in detail in our previous study [16, 17].

### Treatment

First-line immunotherapy in the acute stage was intravenous methylprednisolone (IVMP) combined with intravenous immunoglobulin (IVIG) treatment. Rituximab (RTX) treatment was defined as a second-line treatment.

### Statistical analysis

Statistical analysis was performed using SPSS IBM 20.0. Quantitative data with normal distribution was described by mean ± SD, otherwise median with the interquartile range (IQR). Qualitative data were described by frequency and percentage. Person Chi-Square or Fish exact test was used to compare the qualitative data. Quantitative data with normal distribution were compared using the independent t test, otherwise using Mann-Whitney U test. The p-value < 0.05 (two-sided) was considered significant. Figures were graphed using GraphPad Prism 7.01 (GraphPad Software Inc., US).

## Results

### Demographics

A total of 119 children (male: female = 52:67) diagnosed with anti-NMDAR encephalitis were included. All patients were Chinese Han nationality from southern China, with a mean age of onset of 6.8 ± 3.1 years. Two patients had a history of febrile seizures and had not received ASMs. No tumors were found. Detailed clinical information on patients was presented in Table 1.

**Table 1:** Clinical features of children with anti-NMDAR encephalitis (*n* = 119)

Age (years)	6.8 ± 3.1
Sex (male: female)	52:67
Seizure as the initial symptom (n, (%))	39(32.8)
History of febrile illness preceding the symptom onset (n (%))	2(1.7)
Symptoms in the whole course	
Psychiatric disorders (n (%))	105(88.2)
Movement disorders (n (%))	106(89.1)
Seizure (n (%))	89(74.8)
Speech disorders (n (%))	93(78.2)
Sleep disorders (n (%))	94(79.0)
Consciousness disorder (n (%))	42(35.3)
Dysautonomia (n (%))	39(32.8)
Relapse (n (%))	18(15.1)
Interval from onset to diagnosis (d, median (IQR))	19(14,25)
First hospital stays onset (d, median (IQR))	26(21,34)
Course of IVIG treatment (median (IQR))	2(1,2)
Course of IVMP treatment (median (IQR))	1(1,1)
RTX treatment (n (%))	15(12.6)
mRS before immunotherapy (median (IQR))	4(3,4)
mRS after immunotherapy (median (IQR))	2 (1,2)
Serum anti-NMDAR antibody titers (median (IQR))	1:32 (1:10,1:100)
CSF WBC count (median (IQR), x10^6^/L)	17(8,39)
CSF protein level (median (IQR), g/L)	0.26 (0.21,0.33)
CSF anti-NMDAR antibody titers (median (IQR))	1:10 (1:10, 1:32)
Brain MRI abnormality (n (%))	47(39.5)

CSF: cerebrospinal fluid; IQR: interquartile range; IVIG: intravenous immunoglobulin; IVMP: intravenous methylprednisolone; MRI: magnetic resonance imaging; mRS: modified Rankin Scale; RTX: rituximab

### Clinical characteristics of seizures in the acute stage

**Fig. 1:**
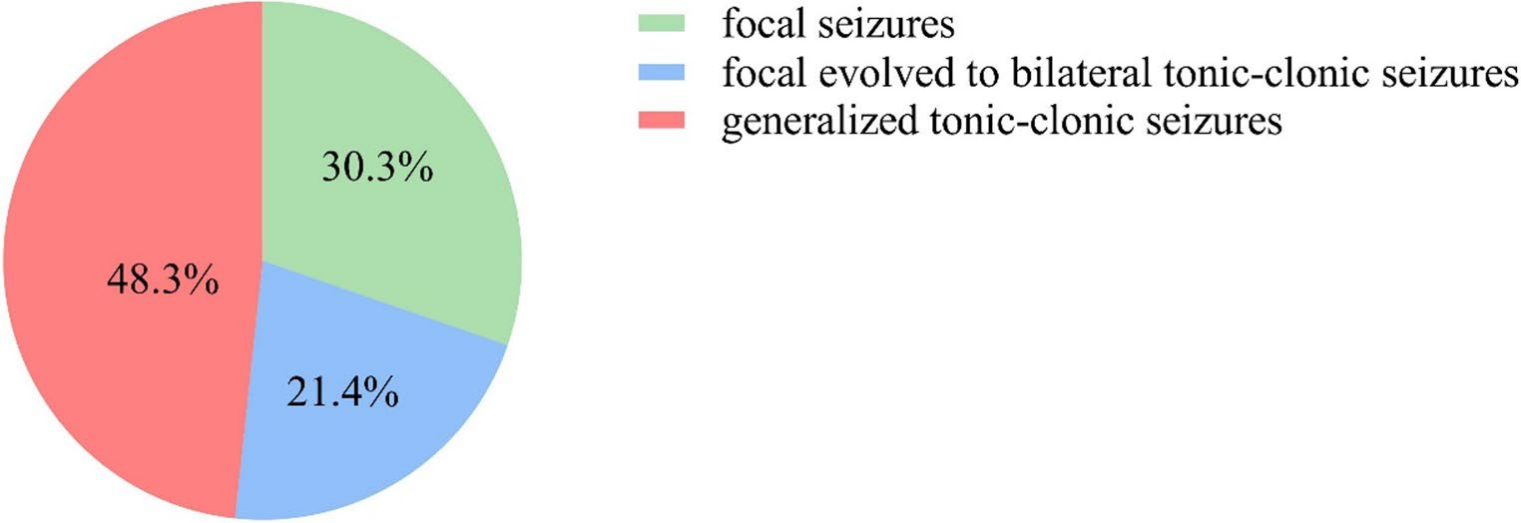
The seizure type classification of patients

**Fig. 2:**
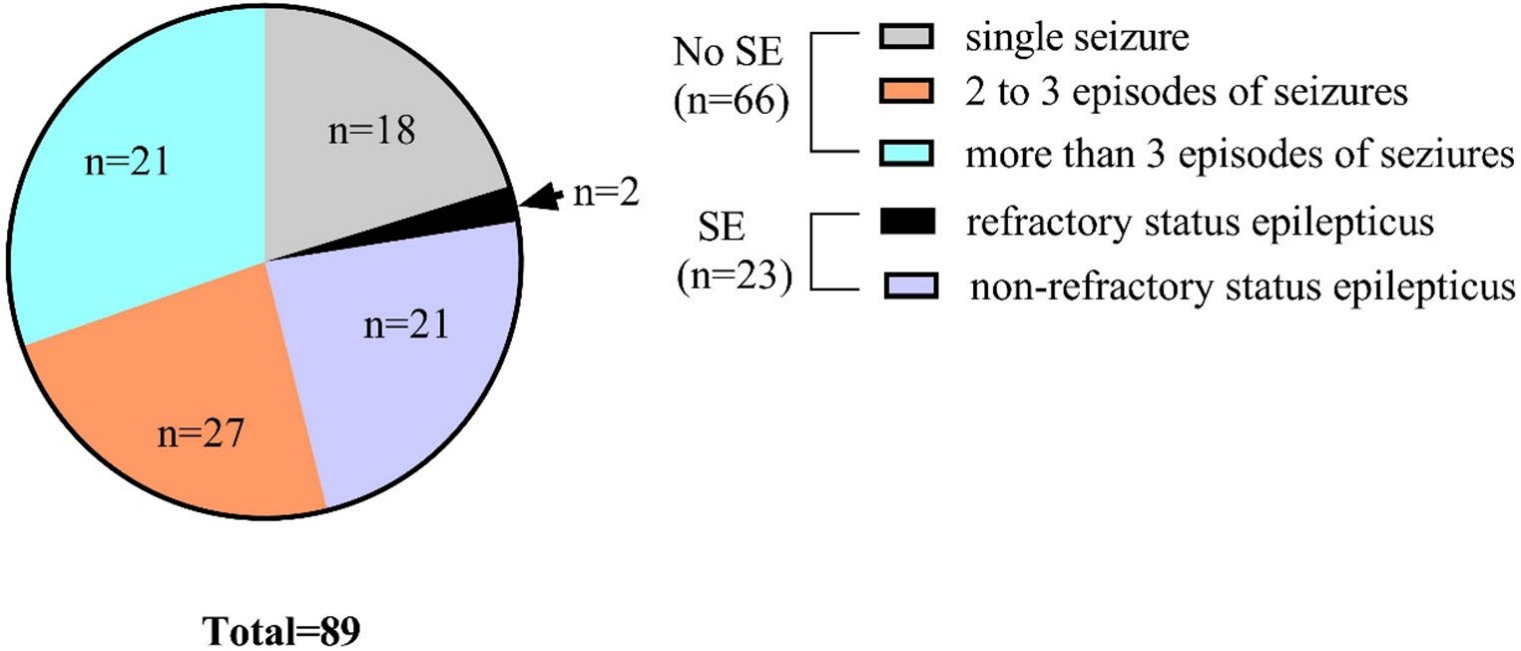
The distribution of seizure episodes in non-status epilepticus patients and types of status epilepticus in status epilepticus patients

32.8% (39/119) of patients presented with seizures as initial symptoms. In total, 74.8% (89/119) of patients experienced seizures during the acute stage, with generalized tonic-clonic seizures (48.3%, 43/89) being the most common type, followed by focal seizures (30.3%, 27/89) and focal to bilateral tonic-clonic seizures (21.4%, 19/89) (more details in Fig. 1). The relapse rate in patients who experienced seizures during the acute stage was 19.1% (17/89), which was significantly higher than the 3.3% (1/30) relapse rate in patients without seizures (Fisher’s exact test, *P* = 0.041). No statistical difference was found in the proportion of presenting seizures between boys and girls (78.8% in boys VS 71.6% in girls, Chi-squared test, χ2 = 0.806, *P* = 0.369). Among these patients, 25.8% (23/89) experienced SE, including 2 cases of refractory SE and 21 cases of non-refractory SE (more details in Fig. 2). The most common type was generalized tonic-clonic seizures, occurring in 73.9% (17/23) of SE cases, followed by focal seizures in four patients and nonconvulsive SE in two patients. In the remaining 74.2% (66/89) of patients without SE, 40.9% (27/66) had more than 3 episodes of seizures, 31.8% (21/66) had 2 to 3 episodes, and 27.3% (18/66) had only a single seizure episode (more details in Fig. 2). The mean NHS3 score of 89 patients with seizures was 7.9 ± 0.9. A comparison of clinical variables with seizure frequency in the acute stage were presented in Table 2. Compared with patients with three or fewer episodes of seizures during the acute stage, patients with more than three episodes of seizures had higher mRS scores before immunotherapy, required longer hospital stays and more courses of IVMP or IVIG treatment and were more likely to require RTX treatment (more details in Table 2).

**Table 2:** Comparison of clinical variables with different seizure frequency in the acute stage

	Total(*n* = 119)	Seizure episodes ≤ 3 during the the acute stage (*n* = 69)	Seizure episodes > 3 during the the acute stage (*n* = 50)	*P*
Age (mean ± SD, years)	6.8 ± 3.1	7.4 ± 2.9	7.0 ± 3.0	0.06^a^
Sex (female, (n, %))	67(56.30)	42(60.9)	25(50.0)	0.238^b^
Hospital day(d, median (IQR))	26(21,34)	23.0(20.0,29.0)	28(23,38)	0.041^c^
Interval from onset to diagnosis (d, median (IQR))	19(14, 25)	17.0(15.0,20.0)	22.5(19.0,30.0)	0.276^c^
CSF WBC(median (IQR), x10^6^/L)	17(8, 39)	15.0(8.0,23.0)	22.5(8.0,70.0)	0.252^c^
CSF protein (median (IQR), g/L)	0.26(0.21,0.33)	0.28(0.23,0.33)	0.28(0.21,0.31)	0.877^c^
Serum NMDAR antibody titers, (median (IQR))	1:32(1:32, 1:100)	1:32(1:10, 1:320)	1:32(1:10, 1:100)	0.426^c^
CSF NMDAR antibody titers (median (IQR))	1:10(1:10, 1:32)	1:32(1:3.2, 1:32)	1:32(1:10, 1:32)	0.925^c^
Follow-up period (m, median (IQR))	16.5(12.0, 27.8)	15.0(9.0,29.0)	19.5(11.0,29.0)	0.891^c^
mRS before immunotherapy (median (IQR))	4(3,4)	4(3,4)	4(3,5)	0.004^c^
mRS after immunotherapy (median (IQR))	2(1,2)	2(1,2)	2(1,2)	0.08^c^
mRS at the last follow-up (median (IQR))	0(0,1)	0(0,1)	0(0,1)	0.971^c^
IVMP course (median (IQR))	1(1,1)	1(1,1)	1(1,2)	0.003^c^
IVIG course (median (IQR))	2(1, 2)	2(1, 2)	2(1, 2)	0.046^c^
RTX (n, %)	15(12.61)	5(7.2)	10(20.0)	0.039^b^
Abnormal brain MRI (n, %)	47(39.50)	25(36.2)	22(44.0)	0.392^b^
Poor prognosis (n, %)	27(22.7)	12(17.4)	15(30.0)	0.105^b^
Relapse (n, %)	18(15.1)	8(11.6)	10(20.0)	0.219^b^

IQR: inter quartile range; CSF: cerebrospinal fluid; IVIG: intravenous immunoglobulin; IVMP: intravenous methylprednisolone; MRI: magnetic resonance image; mRS: Modified Rankin Scale; NMDAR: N-methyl-D-aspartate receptor; RTX: rituximab; SD: standard deviation WBC: white blood cell. a: independent t test; b: Person Chi-Square; c: Mann-Whitney U test

### EEG findings

All patients in the acute stage had abnormal EEG findings, including background slowing in all patients, epileptic waves in 37.0% (44/119) of patients, and extreme delta brush (EDB) in 8.4% (10/119) of patients. Clinical seizures were observed in 9.2% (11/119) of patients during EEG examination, with the focal origin being the most common manifestation. Electrographic seizures without clinical correlates were observed in 10.9% (13/119) of patients during EEG monitoring.

### Treatment

#### Antiepileptic treatment

Among 89 patients with seizure attacks, 53.9% (48/89) of patients received ASMs. The ASMs used included valproate (VPA) (39.6%, 19/48), levetiracetam (LEV) (45.8%, 22/48), oxcarbazepine (OXC) (22.9%, 11/48), nitrazepam (10.4%, 5/48), lamotrigine (8.3%, 4/48), carbamazepine (CBZ) (2.1%, 1/48) and lacosamide (2.1%, 1/48). 72.9% (35/48) of these patients received monotherapy with LEV being the most commonly used, and 18.8 (9/48) received two ASMs, with VPA combined with LEV being the most commonlycombination, 8.3%(4/48) received three or more ASMs. Regarding the first choice of ASMs for patients, LEV was the most commonly prescribed, followed by VPA, OXC, CBZ. One patient received a ketogenic diet for seizure control despite treatment with a combination of LEV, VPA, and OXC. Unfortunately, ketogenic diet is not effective in this case finally. Side effects of ASMs were rarely reported in patients.

#### Immunotherapy

Except for one patient who died before immunotherapy, 118 patients (99.2%, 118/119) received the first-line immunotherapy, and 15 patients received RTX treatment as the second-line treatment. The median number of IVMP courses was 1 (IQR 1,1) and the median number of IVIG courses was 2 (IQR 1,2). 3.4% (4/118) of patients underwent plasma exchange therapy due to rapid disease progression and poor response to IVMP and IVIG. 12.7% (15/118) of patients were treated with RTX, due to poor response to the first-line immunotherapy. Before RTX treatment, there was no significant difference in NHS3 scores between patients who received RTX and those who did not, both before and after RTX treatment. However, the mRS before RTX treatment was higher in patients who received RTX compared to those who did not (more details seen in Table 3). After RTX treatment, no significant difference in mRS was observed between the two groups (more details seen in Table 3).

**Table 3:** Comparison of clinical variables in the SE and no SE groups

	All patient (*n* = 119)	SE (*n* = 23)	No SE (*n* = 96)	*P*
Age (mean ± SD, years)	6.8 ± 3.1	7.1 ± 2.8	6.7 ± 3.1	0.614^a^
Sex (female, (n, %))	67(56.30)	14(60.87)	53(55.21)	0.623^b^
Hospital day (d, median (IQR))	26(21,34)	28(22,43)	26(20,31)	0.965^c^
Interval from onset to diagnosis (d, median(IQR))	19(14, 25)	25.5(19,30)	20(15,25)	0.038^c^
CSF WBC (median (IQR), x10^6^/L)	17(8, 39)	21.5(10,70)	15(7, 32)	0.218^c^
CSF protein (median (IQR), g/L)	0.26(0.21,0.33)	0.26(0.21,0.52)	0.27(0.22,0.32)	0.974^c^
Serum NMDAR antibody titers (median (IQR))	1:32(1:32, 1:100)	1:32(1:32, 1:100)	1:32(1:32, 1:320)	0.708^c^
CSF NMDAR antibody titers (median (IQR))	1:10(1:10, 1:32)	1:10(1:3.2, 1:32)	1:32(1:10, 1:32)	0.282^c^
Follow-up period (m, median (IQR))	16.5(12.0, 27.8)	20.5(12.5, 32.0)	16(10.0, 27.0)	0.332^c^
mRS before immunotherapy, (median (IQR))	4(3,4)	4.5(4,5)	4(3,4)	0.011^c^
mRS after immunotherapy, (median (IQR))	2(1,2)	2(2,3)	2(1,2)	0.263^c^
mRS at the last follow-up (median (IQR))	0(0,1)	0(0,2)	0(0,1)	0.061^c^
IVIG course (median (IQR))	1(1,1)	1(1,2)	1(1,1)	0.033^c^
IVMP course (median (IQR))	2(1, 2)	2(1, 2)	2(1, 2)	0.553^c^
RTX (n, %)	15(12.61)	4(17.39)	11(11.45)	0.468^d^
Abnormal brain MRI (n, %)	47(39.50)	11(47.82)	36(37.50)	0.363^b^
Poor prognosis (n, %)	27(22.7)	4(18.2)	23(23.7)	0.117^d^
Relapse (n, %)	18(15.1)	5(22.7)	13(13.4)	0.428^d^

IQR: inter quartile range; CSF: cerebrospinal fluid; IVIG: intravenous immunoglobulin; IVMP: intravenous methylprednisolone; MRI: magnetic resonance image; mRS: Modified Rankin Scale; NMDAR: N-methyl-D-aspartate receptor; RTX: rituximab; SD: standard deviation WBC: white blood cell. ^a^: independent t test; ^b^: Chi-squared test; ^c^: Wilcoxon rank-sum test; ^d^: Fisher’s exact test

### Outcome of seizure

In this study, 117 surviving patients were followed for a median of 16 months (IQR 10, 27 months) after discharge. Two patients died, both of whom had experienced seizures. Among the 89 patients who had seizures in the acute stage, 23.6% (21/89) achieved seizure remission without ASM treatment before the initiation of immunotherapy, and 22.5% (20/89) achieved remission after immunotherapy without requiring ASM throughout the disease course. Among the remaining 48 patients who received ASM treatment, 95.8% (46/48) achieved seizure remission within one year. Of these, 72.9% (35/48) achieved remission during the acute stage, 12.5% (6/48) within 6 months after the acute stage, and 10.4% (5/48) between 6 and 12 months. Only 4.2% (2/48) continued to experience seizures one year after the acute stage. 25.0% (12/48) of patients discontinued ASM treatment at 6.0 months (IQR 3.5, 15.0 months) after initiation of ASM treatment and no seizures recurred. The remaining 75.0% (36/48) continued ASM therapy until the last follow-up (median 19.5 months, IQR 12.0–30.0 months), 5.6% (2/36) of them continued to experience seizures, despite negative CSF anti-NMDAR antibodies, and one patient continued to have daily seizures despite treatment with three ASMs, a ketogenic diet and monthly IVIG. At the last follow-up, 89.7% (105/117) of patients underwent repeat EEG with normal EEG most commonly seen in 56.2% (59/105), followed by the epileptiform activity or focal slowing with normal EEG background seen in 24.8% (26/105) and generalized slowing including one case with δ brush pattern in the distribution of anterior head in 19.0% (20/105).

### Status epilepticus

Of the 119 patients involved in our study, SE occurred in 23 of 119 (19.3%) patients. Patients with SE showed several differences from those without SE **(**more details in Table 3). The time from onset to diagnosis of anti-NMDAR encephalitis was longer in the SE group. The mRS before immunotherapy was significantly higher in the SE group, and these patients required more courses of IVIG. However, no significant differences in mRS were observed between the two groups after immunotherapy or at the last follow-up, and prognosis was similar in both groups.

## Discussion

Anti-NMDAR encephalitis is the most common type of autoimmune encephalitis [18]. Some clinical features of anti-NMDAR encephalitis differ between pediatric and adult patients; for example, seizures as an initial manifestation are more common in children than in adults [2, 10, 19, 20]. Unlike most published studies focusing on adult patients, our study focused on pediatric anti-NMDAR encephalitis with the largest number of cases in China. In addition, the pathogenic mechanisms and clinical course of anti-NMDAR encephalitis following herpes simplex encephalitis may differ from classical anti-NMDAR encephalitis [21]. Therefore, patients who developed anti-NMDAR encephalitis after herpes simplex encephalitis were exclude in our study.

Based on our previous study [22], this study included more cases. We extended the study and follow-up period, and particularly provided a more detailed summary of onset, auxiliary examination, treatment and prognosis of seizure in children with anti-NMDAR encephalitis. In our study, seizure as the initial symptom was seen in 32.8% of patients with generalized tonic-clonic seizure being the most common type. This ratio falls within the range from 20.3% to 58.1% reported in previous pediatric studies, where generalized onset seizures were also the most common type [2, 3, 20]. Moreover, the frequency of seizures (89/119, 74.8%) during the acute stage in our study was also similar to that observed in other studies [23, 24]. And our study suggests that patients who got more than three seizure episodes during the acute stage needed longer hospital stays and required more actively immunotherapy. However, the frequency of seizures during the acute stage was not associated with poor prognosis.

Monitoring with EEG is crucial for diagnose and treat seizures appropriately. Consistent with previous studies, this study also demonstrated that diffuse slowing was the most common EEG finding in anti-NMDAR encephalitis (100%) [25]. A normal EEG is unusual in patients with anti-NMDAR encephalitis during the acute stage. In 2012, Schmitt et al. first reported that 30.4% (7/23) of adult patients with anti-NMDAR encephalitis had EDB on EEG, which was considered a potential characteristic EEG change for anti-NMDAR encephalitis [26]. Two studies focusing on pediatric anti-NMDAR encephalitis from China showed that the prevalence of EDB was approximately 7% and 16.1%, respectively. In our study, EDB was observed in 8.4% (10/119) of patients, suggesting that EDB may be less common in children with anti-NMDAR encephalitis than in adults.

Overall, evidence or guidelines for ASM treatment in anti-NMDAR encephalitis are lacking. Mechanistically, immunotherapy and tumor removal treatment are causal treatments for anti-NMDAR encephalitis, whereas ASMs serve as symptomatic treatments. Seizure freedom is not always achieved with immunotherapy alone, and ASMs are sometimes required. We found 23.6% (21/89) of patients with seizures in the acute stage achieved seizure remission without ASM treatment before initiation of immunotherapy, and 22.5% (20/89) of these patients achieved seizure remission after initiation of immunotherapy, and ASMs were not initiated during the whole disease course. In our study, 53.9% of patients with seizures were treated with ASMs. LEV and VPA were commonly used due to their rapid actions and the ability to be used without gradually increasing the dosage. The side effects of ASMs were rarely reported. The proportion of patients treated with ASMs and the types of ASMs used were similar to other studies [2, 3]. However, a recent study showed that LEV could induce or exaggerate serious behavioral disorders in adult anti-NMDAR patients [27]. Whether this is the case in children is unclear, and thus, the appropriate ASMs for seizures in pediatric anti-NMDAR encephalitis require further investigation. In addition, CBZ or OXC is not often chosen due to the risk of severe allergic rash, which are more likely to occur in the Asian population with specific pro-inflammation human leukocyte antigen (HLA) types, such as HLA-B1502 [28–30]. For safety reasons, parents of children are often reluctant to choose CBZ or OXC. Meanwhile, HLA-B 1502 testing has a long turnaround time and is relatively expensive, making OXC or CBZ less likely to be the first-line choice. Prospective studies are needed to compare different ASMs treatments for seizures in anti-NMDAR encephalitis after the acute stage.

After the acute stage of anti-NMDAR encephalitis, the continued use of ASMs is debatable. In our study, we found that 95.8% (46/48) of patients receiving ASM treatment achieved seizure remission within one year after the acute stage, while only 4.2% (2/48) of patients continued to experience seizures one year after the acute stage. Similarly, Liu et al. found that seizure freedom was achieved within 2 years in all anti-NMDAR encephalitis patients, and 80% of these patients had their last seizure within 6 months [2]. In our study, 25.0% (12/48) of patients discontinued ASM treatment at 6.0 months (IQR 3.5, 15.0 months) after initiation of ASM treatment and no seizures recurred. While 75.0% (36/48) of patients were still receiving ASMs treatment at the last follow-up, 5.6% (2/36) of them continued to experience seizures, despite their CSF anti-NMDAR antibodies having already turned negative. Zhang et al. analyzed the relationship between cranial MRI abnormalities and seizures in children with anti-NMDAR encephalitis [31]. Patients in their study were dived into non-lesion group, lesions in the limbic system group and the neocortical lesion group. They found only patients in the neocortical cortex lesion group continued to experience seizures 1 year after hospital discharge, suggesting that neocortex involvement is more likely to cause epilepsy. In our study, the median duration of ASM treatment was 14.5 months (IQR 7.8, 27.5 months). In Qu et al.’ study about seizure characteristics in pediatric anti-NMDAR encephalitis, the duration of ASMs treatment was 3.58 ± 1.08 months in the short-term seizures group and 8.40 ± 1.14 months in the persistent seizures group, and no patients experienced seizures after cessation of ASMs treatment [3]. In summary, for patients with anti-NMDAR encephalitis, continued used of ASMs after the acute stage of anti-NMDAR encephalitis is needed, but perhaps long-term and sustained administration may not be necessary.

SE is common observed in the acute stage of anti-NMDAR encephalitis [14, 32]. In a cohort of 109 patients with anti-NMDAR encephalitis, 88.1% of whom were older than 18 years old, the incidence of SE was 50%. While in Wang et al.’s study, which involved patients older than 14, SE was observed in 15.7% (13/83) of patients [14]. Besides, in a study of pediatric anti-NMDAR encephalitis, SE was seen in 43.5% (27/62) of patients [3]. In our study, 19.3% (23/119) of patients had SE. The ratios of SE in anti-NMDAR varied in different studies. Furthermore, we found that SE patients had a longer interval from onset to diagnosis than those without SE. Whether the differences in SE frequency between studies were caused by the different diagnosis intervals in different studies was unknown. The correlation between SE and the prognosis of anti-NMDAR encephalitis remains controversial. Huang et al.’s study showed that adults with anti-NMDAR encephalitis exhibit worse outcomes than children, primarily due to SE [33]. However, another systematic review on the prognosis of autoimmune encephalitis, including anti-NMDAR encephalitis, showed that SE was unlikely to have significant prognostic value in adult cases [34]. Our findings suggest that SE in patients with anti-NMDAR encephalitis may not be associated with poor prognosis, but such patients require more courses of IVIG treatment in the acute stage. It remains controversial since there are few studies focused on children. Further prospective investigations assessing the correlation between SE and prognosis are needed.

For anti-NMDAR encephalitis, several studies have suggested that clinical severity may be associated with CSF anti-NMDAR antibody titers, and that antibody levels may complement clinical assessment [35, 36]. In most cases, when antibodies are rapidly suppressed or removed, synaptic density recovers and clinical symptoms improve markedly [37]. In wang et al.‘s study, strong positive anti-NMDAR antibody titers were more frequently observed in patients with SE than in those without SE [14]. However, in our study, although the mRS score before immunotherapy was higher in the SE group than in the non-SE group, no significant difference in CSF anti-NMDAR antibody titers was detected between the two groups. The neurobiology of anti-NMDAR encephalitis is more complex than a simple model of NMDAR hypofunction. Anti-NMDAR antibodies can act as both NMDAR antagonists and agonists, and their effects may vary depending on receptor conformation and subtype [1]. Besides, the progressive decrease of NMDAR caused by the anti-NMDAR antibodies and then gradual restoration of NMDAR after a decline in antibody titer result in the multistage process of illness of anti-NMDAR encephalitis [36]. The different times of antibody tests may also impact the association of antibody titer and clinical severity as well as SE analysis.

## Conclusions

Seizures are common in pediatric anti-NMDAR encephalitis; patients with more than three seizure episodes had higher mRS pre-immunotherapy and required more intensive treatment; Approximately half of the patients required ASMs, with 95.8% achieving remission within one year. The relapse rate was significantly higher in patients with seizures during the acute stage compared to those without seizures.Patients with SE had higher mRS pre-immunotherapy and required more IVIG courses, but their prognosis was similar to that of non-SE patients.

## Data Availability

The datasets generated or analyzed during the current study are not publicly available due the data repository in our hospital is still under construction but are available from the corresponding author on reasonable request.

## **Abbreviations**

ASM: anti-seizure medication
CBZ: carbamazepine
CSF: cerebrospinal fluid
EDB: extreme delta brush
EEG: electroencephalograph
HLA: human leukocyte antigen
IQR: interquartile rangeIQR
IVIG: intravenous immunoglobulin
IVMP: intravenous methylprednisolone
LEV: levetiracetam
MRI: magnetic resonance imaging
mRS: modified Rankin Scale
NHS3: Chalfont-National Hospital seizure severity scale
NMDAR: N-methyl-D-aspartate receptor
OXC: oxcarbazepine
RTX: rituximab
SE: status epilepticus
VPA: valproate

## References

[1] Lynch DR, Rattelle A, Dong YN, Roslin K, Gleichman AJ, Panzer JA (2018) Anti-NMDA receptor encephalitis: clinical features and basic mechanisms. Adv Pharmacol 82:235–260. 10.1016/bs.apha.2017.08.005 29413523 10.1016/bs.apha.2017.08.005

[2] Liu X, Yan B, Wang R, Li C, Chen C, Zhou D, Hong Z (2017) Seizure outcomes in patients with anti-NMDAR encephalitis: A follow-up study. Epilepsia 58(12):2104–2111. 10.1111/epi.13929 29098690 10.1111/epi.13929

[3] Qu XP, Vidaurre J, Peng XL, Jiang L, Zhong M, Hu Y (2020) Seizure Characteristics, Outcome, and risk of epilepsy in pediatric Anti-N-Methyl-d-Aspartate receptor encephalitis. Pediatr Neurol 105:35–40. 10.1016/j.pediatrneurol.2019.11.011 31917096 10.1016/j.pediatrneurol.2019.11.011

[4] Dalmau J, Armangué T, Planagumà J, Radosevic M, Mannara F, Leypoldt F, Geis C, Lancaster E, Titulaer MJ, Rosenfeld MR, Graus F (2019) An update on anti-NMDA receptor encephalitis for neurologists and psychiatrists: mechanisms and models. Lancet Neurol 18(11):1045–1057. 10.1016/s1474-4422(19)30244-3 31326280 10.1016/S1474-4422(19)30244-3

[5] Ren C, Zhang W, Ren X, Li J, Ding C, Wang X, Ren H, Fang F (2021) Clinical features and outcomes of Anti-N-Methyl-d-Aspartate receptor encephalitis in infants and toddlers. Pediatr Neurol 119:27–33. 10.1016/j.pediatrneurol.2021.02.009 33838580 10.1016/j.pediatrneurol.2021.02.009

[6] Dalmau J (2016) NMDA receptor encephalitis and other antibody-mediated disorders of the synapse: the 2016 Cotzias lecture. Neurology 87(23):2471–2482. 10.1212/wnl.0000000000003414 27920282 10.1212/WNL.0000000000003414 PMC5177671

[7] Cramer JA (2001) J. <>French Quantitative assessment of seizure severity for clinical trials: a review of approaches to seizure components. Epilepsia 42 1 119–129 10.1046/j.1528-1157.2001.19400.x 11207795 10.1046/j.1528-1157.2001.19400.x

[8] Dalmau J, Lancaster E, Martinez-Hernandez E, Rosenfeld MR, Balice-Gordon R (2011) Clinical experience and laboratory investigations in patients with anti-NMDAR encephalitis. Lancet Neurol 10(1):63–74. 10.1016/s1474-4422(10)70253-2 21163445 10.1016/S1474-4422(10)70253-2 PMC3158385

[9] Graus F, Titulaer MJ, Balu R, Benseler S, Bien CG, Cellucci T, Cortese I, Dale RC, Gelfand JM, Geschwind M, Glaser CA, Honnorat J, Hoftberger R, Iizuka T, Irani SR, Lancaster E, Leypoldt F, Pruss H, Rae-Grant A, Reindl M, Rosenfeld MR, Rostasy K, Saiz A, Venkatesan A, Vincent A, Wandinger KP, Waters P, Dalmau J (2016) A clinical approach to diagnosis of autoimmune encephalitis. Lancet Neurol 15(4):391–404. 10.1016/s1474-4422(15)00401-9 26906964 10.1016/S1474-4422(15)00401-9 PMC5066574

[10] Titulaer MJ, McCracken L, Gabilondo I, Armangué T, Glaser C, Iizuka T, Honig LS, Benseler SM, Kawachi I, Martinez-Hernandez E, Aguilar E, Gresa-Arribas N, Ryan-Florance N, Torrents A, Saiz A, Rosenfeld MR, Balice-Gordon R, Graus F, Dalmau J (2013) Treatment and prognostic factors for long-term outcome in patients with anti-NMDA receptor encephalitis: an observational cohort study. Lancet Neurol 12(2):157–165. 10.1016/s1474-4422(12)70310-1 23290630 10.1016/S1474-4422(12)70310-1 PMC3563251

[11] Fisher RS, Cross JH, French JA, Higurashi N, Hirsch E, Jansen FE, Lagae L, Moshé SL, Peltola J, Roulet Perez E, Scheffer IE, Zuberi SM (2017) Operational classification of seizure types by the international league against epilepsy: position paper of the ILAE commission for classification and terminology. Epilepsia 58(4):522–530. 10.1111/epi.13670 28276060 10.1111/epi.13670

[12] Zhang Y, Deng C, Zhu L, Ling L (2020) Predisposing factors and prognosis of status epilepticus in patients with autoimmune encephalitis. Med (Baltim) 99(13):e19601. 10.1097/md.0000000000019601 10.1097/MD.0000000000019601 PMC7220189 32221081

[13] Trinka E, Cock H, Hesdorffer D, Rossetti AO, Scheffer IE, Shinnar S, Shorvon S, Lowenstein DH (2015) A definition and classification of status epilepticus–Report of the ILAE task force on classification of status epilepticus. Epilepsia 56(10):1515–1523. 10.1111/epi.13121 26336950 10.1111/epi.13121

[14] Wang X, Wan J, Wei Z, Song C, Kang X, Du F, Jiang W, Yang F (2022) Status epilepticus in patients with Anti-NMDAR encephalitis requiring intensive care: A Follow-Up study. Neurocrit Care 36(1):192–201. 10.1007/s12028-021-01283-4 34286465 10.1007/s12028-021-01283-4

[15] Arya R, Rotenberg A (2019) Dietary, immunological, surgical, and other emerging treatments for pediatric refractory status epilepticus. Seizure 68:89–96. 10.1016/j.seizure.2018.09.002 30245007 10.1016/j.seizure.2018.09.002

[16] Hou C, Wu W, Tian Y, Zhang Y, Zhu H, Zeng Y, Peng B, Zheng K, Li X, Chen W (2020) Clinical analysis of anti-NMDAR encephalitis combined with MOG antibody in children. Mult Scler Relat Disord 42:102018. 10.1016/j.msard.2020.102018 32234601 10.1016/j.msard.2020.102018

[17] Chen L, Chen C, Zhong X, Sun X, Zhu H, Li X, Yang H, Shu Y, Chang Y, Hu X, Lu Z, Peng L, Qiu W (2018) Different features between pediatric-onset and adult-onset patients who are seropositive for MOG-IgG: A multicenter study in South China. J Neuroimmunol 321:83–91. 10.1016/j.jneuroim.2018.05.014 29957392 10.1016/j.jneuroim.2018.05.014

[18] Gurrera RJ (2019) Recognizing psychiatric presentations of anti-NMDA receptor encephalitis in children and adolescents: A synthesis of published reports. Psychiatry Clin Neurosci 73(5):262–268. 10.1111/pcn.12821 30653785 10.1111/pcn.12821

[19] Roliz A, Shah Y, Morse A, Troester M, Lynch R, Pickle J, Karkare S, Fernandez-Carbonell C, Kothare S (2021) Clinical features of paediatric and adult autoimmune encephalitis: A multicenter sample. Eur J Paediatr Neurol 30:82–87. 10.1016/j.ejpn.2021.01.001 33461084 10.1016/j.ejpn.2021.01.001

[20] Zhang L, Wu MQ, Hao ZL, Chiang SM, Shuang K, Lin MT, Chi XS, Fang JJ, Zhou D, Li JM (2017) Clinical characteristics, treatments, and outcomes of patients with anti-N-methyl-d-aspartate receptor encephalitis: A systematic review of reported cases. Epilepsy Behav 68:57–65. 10.1016/j.yebeh.2016.12.019 28109991 10.1016/j.yebeh.2016.12.019

[21] Sun B, Ramberger M, O’Connor KC, Bashford-Rogers RJM, Irani SR (2020) The B cell immunobiology that underlies CNS autoantibody-mediated diseases. Nat Rev Neurol 16(9):481–492. 10.1038/s41582-020-0381-z 32724223 10.1038/s41582-020-0381-z PMC9364389

[22] Li X, Hou C, Wu WL, Liang H, Zheng K, Zhang Y, Zeng Y, Chen L, Zhu H, Tian Y, Gao Y, Peng B, Yang S, Wang X, Ning S, Liao Y, Lin H, Chen WX (2021) Pediatric anti-N-methyl-d-aspartate receptor encephalitis in Southern china: analysis of 111 cases. J Neuroimmunol 352:577479. 10.1016/j.jneuroim.2021.577479 33486307 10.1016/j.jneuroim.2021.577479

[23] Zhang J, Ji T, Chen Q, Jiang Y, Cheng H, Zheng P, Ma W, Lei T, Zhang Y, Jin Y, Wei C, Wu Y, Chang X, Bao X, Zhang Y, Xiong H, Ji X, Feng S, Ren H, Yang J, Jiang Y (2019) Front Neurol 10:906. Pediatric Autoimmune Encephalitis: Case Series From Two Chinese Tertiary Pediatric Neurology Centers10.3389/fneur.2019.00906 31507515 10.3389/fneur.2019.00906 PMC6714292

[24] Dalmau J, Gleichman AJ, Hughes EG, Rossi JE, Peng X, Lai M, Dessain SK, Rosenfeld MR, Balice-Gordon R, Lynch DR (2008) Anti-NMDA-receptor encephalitis: case series and analysis of the effects of antibodies. Lancet Neurol 7(12):1091–1098. 10.1016/s1474-4422(08)70224-2 18851928 10.1016/S1474-4422(08)70224-2 PMC2607118

[25] da Silva-Júnior FP, Castro LH, Andrade JQ, Bastos CG, Moreira CH, Valério RM, Jorge CL, Marchiori PE, Nitrini R, Garzon E (2014) Serial and prolonged EEG monitoring in anti-N-Methyl-d-Aspartate receptor encephalitis. Clin Neurophysiol 125(8):1541–1544. 10.1016/j.clinph.2014.01.001 24457136 10.1016/j.clinph.2014.01.001

[26] Schmitt SE, Pargeon K, Frechette ES, Hirsch LJ, Dalmau J, Friedman D (2012) Extreme delta brush: a unique EEG pattern in adults with anti-NMDA receptor encephalitis. Neurology 79(11):1094–1100. 10.1212/WNL.0b013e3182698cd8 22933737 10.1212/WNL.0b013e3182698cd8 PMC3525298

[27] de Bruijn M, van Sonderen A, van Coevorden-Hameete MH, Bastiaansen AEM, Schreurs MWJ, Rouhl RPW, van Donselaar CA, Majoie M, Neuteboom RF, Sillevis Smitt PAE, Thijs RD, Titulaer MJ (2019) Evaluation of seizure treatment in anti-LGI1, anti-NMDAR, and anti-GABA(B)R encephalitis. Neurology 92(19):e2185–e2196. 10.1212/wnl.0000000000007475 30979857 10.1212/WNL.0000000000007475 PMC6537134

[28] Grover S, Kukreti R (2014) HLA alleles and hypersensitivity to carbamazepine: an updated systematic review with meta-analysis. Pharmacogenet Genomics 24(2):94–112. 10.1097/fpc.0000000000000021 24336023 10.1097/FPC.0000000000000021

[29] Zhang Y, Wang J, Zhao LM, Peng W, Shen GQ, Xue L, Zheng XX, He XJ, Gong CY, Miao LY (2011) Strong association between HLA-B*1502 and carbamazepine-induced Stevens-Johnson syndrome and toxic epidermal necrolysis in Mainland Han Chinese patients. Eur J Clin Pharmacol 67(9):885–887. 10.1007/s00228-011-1009-4 21424386 10.1007/s00228-011-1009-4

[30] Hu FY, Wu XT, An DM, Yan B, Stefan H, Zhou D (2011) Pilot association study of oxcarbazepine-induced mild cutaneous adverse reactions with HLA-B*1502 allele in Chinese Han population. Seizure 20(2):160–162. 10.1016/j.seizure.2010.11.014 21169036 10.1016/j.seizure.2010.11.014

[31] Zhang J, Sun J, Zheng P, Feng S, Yi X, Ren H, Chen Q (2021) Clinical characteristics and Follow-Up of seizures in children with Anti-NMDAR encephalitis. Front Neurol 12:801289. 10.3389/fneur.2021.801289 35069429 10.3389/fneur.2021.801289 PMC8766335

[32] Lin J, Xiang Q, Liu X, Li J (2022) Risk factors and prognosis in patients with Anti-N-Methyl-D-Aspartate receptor encephalitis requiring prolonged mechanical ventilation. Front Neurol 13:814673. 10.3389/fneur.2022.814673 35222249 10.3389/fneur.2022.814673 PMC8863869

[33] Huang Q, Wu Y, Qin R, Wei X, Ma M (2016) Clinical characteristics and outcomes between children and adults with anti-N-Methyl-D-Aspartate receptor encephalitis. J Neurol 263(12):2446–2455. 10.1007/s00415-016-8282-1 27632180 10.1007/s00415-016-8282-1

[34] Broadley J, Seneviratne U, Beech P, Buzzard K, Butzkueven H, O’Brien T, Monif M (2019) Prognosticating autoimmune encephalitis: A systematic review. J Autoimmun 96:24–34. 10.1016/j.jaut.2018.10.014 30595145 10.1016/j.jaut.2018.10.014

[35] Zhang Y, Liu G, Jiang M, Chen W, He Y, Su Y (2018) Clinical characteristics and prognosis of severe Anti-N-methyl-D-aspartate receptor encephalitis patients. Neurocrit Care 29(2):264–272. 10.1007/s12028-018-0536-6 29651625 10.1007/s12028-018-0536-6

[36] Gresa-Arribas N, Titulaer MJ, Torrents A, Aguilar E, McCracken L, Leypoldt F, Gleichman AJ, Balice-Gordon R, Rosenfeld MR, Lynch D, Graus F, Dalmau J (2014) Antibody titres at diagnosis and during follow-up of anti-NMDA receptor encephalitis: a retrospective study, Lancet Neurol 13(2) 167 – 77. 10.1016/s1474-4422(13)70282-5 10.1016/S1474-4422(13)70282-5 PMC4006368 24360484

[37] Fukata M, Yokoi N, Fukata Y (2018) Neurobiology of autoimmune encephalitis. Curr Opin Neurobiol 48:1–8. 10.1016/j.conb.2017.07.012 28829986 10.1016/j.conb.2017.07.012

